# Visual fatigue caused by watching 3DTV: an fMRI study

**DOI:** 10.1186/1475-925X-14-S1-S12

**Published:** 2015-01-09

**Authors:** Chunxiao Chen, Jing Wang, Kun Li, Yuping Liu, Xin Chen

**Affiliations:** 1 Department of Biomedical Engineering, Nanjing University of Aeronautics & Astronautics, Nanjing, 210016, China; 2 Department of Radiology, Guangdong Province Traditional Chinese Medical Hospital, Guangzhou, 510006, China

**Keywords:** Visual fatigue, fMRI, 3DTV, Stereoscopic images

## Abstract

The objective of this study is to observe the visual fatigue caused by watching 3DTV using the method of functional magnetic resonance imaging (fMRI). The data of fMRI during three kinds of visual stimulation tasks were obtained from twenty subjects. At first, blood-oxygen-level dependent (BOLD) signal changes during stimuli of checkerboard task were compared before and after one-hour watching 3D/2DTV, and subjective evaluation was conducted based on the questionnaire simultaneously. Then 3D and 2D images were used to stimulate healthy individuals to measure brain activities that correlated with stereoscopic vision. Finally, the relationship between front or back depth of field images and visual fatigue was investigated. The results reveal that the 3D group shows more significant differences of brain activities in BA8, BA17, BA18 and BA19 than the 2D group during the checkerboard stimulation. BA5, BA6, BA7 and BA8 were testified to have close relationship with stereoscopic perception via the 2D/3D images stimulation. Furthermore, the front depth of field image was proven to impose a more serious impact on visual fatigue than the back one. These conclusions are useful for healthy and reasonable 3DTV watching as well as properly designing of 3D scenes.

## Introduction

Three-dimensional television (3DTV) is perceived as an innovative invention, since this piece of new technology boosts the viewing experience by showing stereoscopic images. Nevertheless, some viewers have experienced a series of physical discomfort after a prolonged watching of 3DTV, such as severe headache, diplopia and esotropia [1]. Among all of these discomforts, visual fatigue is the most adverse reaction [2]. During the stereoscopic display, the observer's left and right eye will receive the 2D offset image separately. These two images will then combine in the observer's brain to give the perception of 3D depth [3]. The uncoupling of convergence and accommodation that reduces one's ability to fuse the binocular stimulus is the main cause of visual fatigue. Moreover, serious visual fatigue can lead to changes in fusional vergence limit and its hysteresis, persistent headaches, muscle pain in the head, neck and upper back, as well as long term problems like myopia [4,5].

Researchers have paid more and more attention on the potential risks to human health brought by watching 3DTV. In reference [6], the visual fatigue was compared between watching high-definition TV and stereo TV with the method of Single-Stimulus Continuous Quality Evaluation (SSCQE), and it indicated that the degree of visual fatigue was correlated with the depth of information. However, visual fatigue is an essentially subjective feeling based on the personal sense [7]. In order to establish the health standard for 3DTV and develop the 3DTV fatigue countermeasure, objective and quantitative evaluation criteria should be built by the physiological signal analysis. Various methods, such as electrooculogram (EOG), electrocardiogram (ECG) and electroencephalogram (EEG) have been applied in the recent studies of visual fatigue. In reference [8], EEG was used to detect a typical of P300 by event-related potential (ERP) during watching stereo images. Signal apparently became clear after the stimulation of 700 milliseconds, which turned out that there was a correlation between visual fatigue and viewing stereo images. In our previous study, the energy of EEG in the four wavebands, namely alpha, beta, theta, delta, and four fatigue related algorithms were compared after watching 2DTV and 3DTV. Combining with the subjective evaluation, we gave an objective indicator to evaluate the level of fatigue [9]. In recent years, functional magnetic resonance imaging (fMRI) has become a powerful method in brain mapping research due to its non-invasion, non-radiation and high spatial resolution [10]. The brain regions activated by specific stimuli can be detected using the blood-oxygen-level dependent (BOLD) contrast. For instance, fMRI demonstrates the association between the feelings of mental fatigue and the BOLD responses by a mentally fatiguing cognitive task [11]. Gaebler M [12] compares the brain activity of subjects when freely watching the same movie in both 2D and 3D, and observes significantly higher intersubject correlations with cortical networks when watching the 3D version. Based on our knowledge, there is no report using fMRI to study the visual fatigue caused by stereoscopic viewing.

In this study, we have investigated the relationship between the visual fatigue and BOLD responses during watching 3DTV. In section 2, checkerboard stimuli and classical block stimuli schemes are illustrated while watching 3D/2DTV. In section 3, subjective questionnaire and fMRI response before and after watching 3D/2DTV are given. Finally, the relationship between visual fatigue and BOLD response areas is discussed in section 4, and our preliminary results suggest that prolonged watching of 3DTV could result in more serious visual fatigue than watching 2DTV.

## Materials and methods

### Participants

Forty participants, aged 24-30 (mean: 26 years old), were randomly recruited from Zhongshan University. Eight 3D-movie clips with four levels of depth of field (1/2 in front of and 1/2 behind the focus point) were displayed to ensure that they had normal stereoscopic sensitivity. In the meantime, they do not have medical contraindications such as severe concomitant disease, alcoholism, drug abuse, as well as psychological or intellectual problems which are likely to limit compliance [13]. After screening, twenty participants were selected. The experimental purposes and procedures have been informed to everyone in advance, and the study has been approved by the local ethics committee. Participants are required to keep in a good condition both physically and mentally, and have good sleep the previous day. To avoid low blood sugar levels that may affect the experiment results, the subjects are required to have a meal about 1 to 2 hours before experiment. They watch 3D movie on the first day and 2D movie under the same conditions the next day. Each experiment lasts approximately 2 hours, and all participants have received 300 RMB for participation.

### Experimental design

#### Stimuli of checkerboard

**Figure 1:**
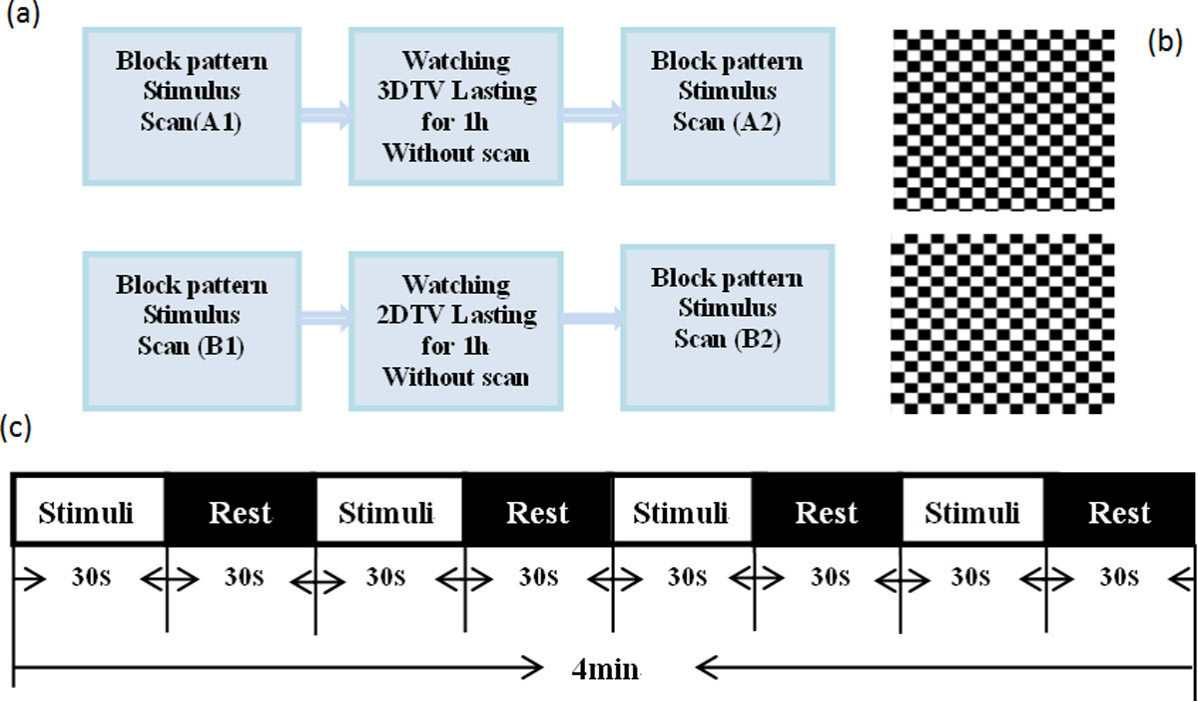
**Illustration for checkerboard stimuli**. (a) FMRI scans before and after watching 3DTV and 2DTV. (b) Black-white checkerboard. (c) The sequence of checkerboard stimuli and blank screen for rest.

The block design task on checkerboard stimuli is performed before and immediately after watching 2DTV or 3DTV (Figure 1a). Stimulus is black-white checkerboard made by equal-sized squares of black and white colors as showed in Figure 1b. Each small square in the checkerboard changes from white to black and then black to white in 8Hz. The fMRI data are acquired only in the periods of A1, A2, B1 and B2, and no scanning is conducted during the one-hour 2D/3DTV watching. The condition for successive blocks alternates between blank screen for rest and black-white checkerboard for stimulation (Figure 1c). Each block lasts for 30s and the total presentation time is 4min. Subjective questionnaire form (Table 1) is filled by the participant after scanning A2 and B2 immediately. Table 2 defines the description of each fatigue level. 40 Bold data sets (2D and 3D in total) have been collected from 2D group and 3D group, and the changes of brain activation before and after watching 2D/3DTV are analyzed by comparing A1 and A2 as well as B1 and B2 respectively.

**Table 1:** Subjective questionnaire for watching 3DTV.

	Nothing	Mild	Less moderate	Moderate	Less severe	Severe
Headache						
Watery eyes						
Stinging						
Bleary						
Double vision						
Nausea						
Grittiness						
Dizziness						
Eyestrain						
Difficult focusing						
Vomiting						

**Table 2:** Subjective fatigue evaluation.

level	Fatigue state	State characteristics
**0**	No fatigue	Without obvious characteristics
**1**	Mild fatigue	Mild eyestrain and grittiness
**2**	Less moderate fatigue	Moderate or less moderate eyestrain, mild stinging and dizziness
**3**	Moderate fatigue	Moderate or less severe eyestrain, moderate or less moderate stinging, mildly bleary, moderately dizzy, mild and less moderate nausea and headache
**4**	Less severe fatigue	Severe or less severe eyestrain, severe grittiness, moderate or less severe dizziness and nausea
**5**	Severe fatigue	Severe eyestrain, severe dizziness, severe or less severe headache, nausea and vomiting

#### Stimuli of 3D and 2D images

Materials needed: 10 plane images and 20 stereo images, whose depth of field are 1/2 in front of and 1/2 behind the focus point.

**Figure 2:**
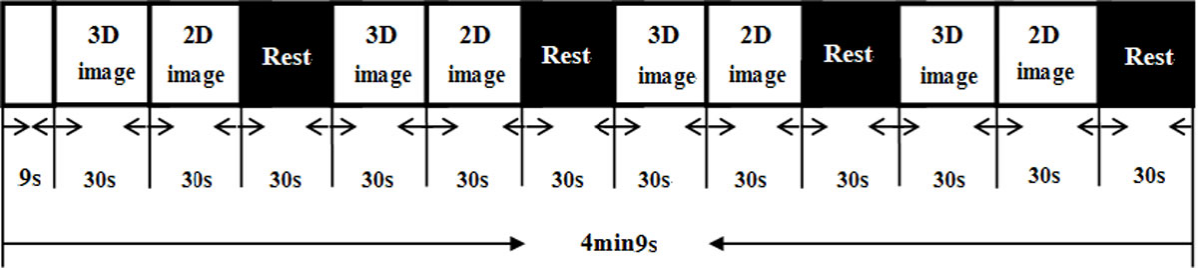
**Illustration of 3D and 2D images stimulus**.

In addition to justify the visual fatigue produced by watching 3D images, this program will also explore the variation of brain activation caused by the depth of field. The scheme of block design is shown in Figure 2. Ten stereoscopic images are displayed one by one, with each lasting for 3 seconds in part A and the same procedure is applied for 2D-images in part B. In part C, the blank screen stimuli is tested as a benchmark/calibration of the brain activation. There is a 9s-blank stimuli at the beginning for preparation, and then three conditions are presented alternately for four times. Therefore, the experiment lasts for 6 min 9s, and 123 acquisitions are obtained. The experiment is repeated twice, one for viewing the stereo images with front depth of the field, and another with back depth of the field.

### MRI acquisition and analysis

Participants lay supinely with their head snugly fixed by a belt and foam pads to minimize head movement, and no participant had head motion more than 2.0mm. Scans were obtained on GE-HDx 3.0T MR system in Guangdong Province Traditional Chinese Medical Hospital. T1-weighted structure images of the whole brain were acquired from FLASH scans. TR = 25 ms; TE = 6 ms; flip angle = 30°; FOV = 240 mm × 240 mm; matrix = 256 × 256; slice thickness = 2 mm. Functional images with asymmetric spin-echo EPI were obtained according to the schemes designed before. TR = 3000 ms; TE = 35 ms; FOV = 240 mm × 240 mm; matrix = 64 × 64 × 36; flip angle = 90°; slice thickness = 4 mm; spatial resolution = 4 mm × 4 mm × 4 mm.

Post-processing and statistical analysis of the fMRI data are carried out by Statistical Parametric Mapping (SPM8, Wellcome Department of Cognitive Neurology, London, UK). The "Realignment" in SPM8 is first used to either retrospectively or prospectively compensate for the effects of motion on the image data. In this experiment, all volumes are realigned to the first volume to correct for artifact induced by head movement between scans. The functional images are registered to the corresponding T1-weighted structural images, normalized to the MNI standard brain, and smoothed with a Gaussian kernel of full width at half maximum (FWHM) 8 mm.

A general linear model (GLM) approach is applied to analysis of fMRI time-series for indicating where the brain has been activated in response to the stimuli [14]. For each single factor, such as checkerboard stimuli before or after watching TV and stimuli of 2D or 3D images with the front and back depth of field, activation maps are calculated using one sample t-test. Cluster detection is performed and the cluster with its size above 5 is determined to be significantly activated (P < 0.05) [15]. Two sample t-test is then run to compare differences in activation between pre- and post-watching TV, stimulation of 2D and 3D images as well as 3D displays with the front depth of field and back depth of field. Activation maps are set by clusters above 10 and the peak activity of P < 0.01 [16]. The anatomical labels of brain activations and their corresponding Brodmann Areas (BA) are found using MNI standard brain.

## Results

### Subjective analysis

**Figure 3:**
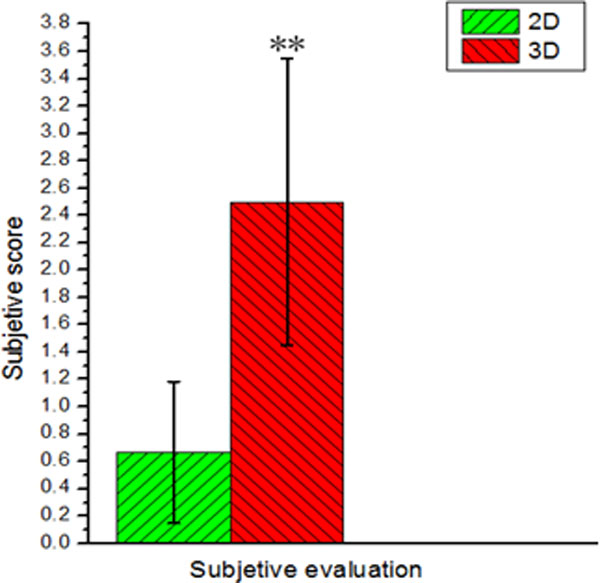
**Comparison of subjective fatigue level caused by watching 2DTV and 3DTV**.

Based on the subjective questionnaire in Table 1 fatigue evaluation was conducted according to the criteria shown in Table 2. The comparison of subjective fatigue level between 2D and 3D groups are shown in Figure 3. Obviously, compared with the 2D group, the mean value of the subjective fatigue level is significantly larger in the 3D group, indicating that continuous watching of 3DTV will lead to more serious visual fatigue.

### Neuron activity

According to different experimental purposes, three studies have been analyzed as the following: 1) checkerboard stimulus performance before and after watching 3D/2DTV; 2) differences of activation between watching 3D and 2D images; 3) differences of activation between watching front depth of field 3D images and back depth of field 3D images.

#### Checkerboard stimulus performance before and after watching 3D/2D TV

**Figure 4:**
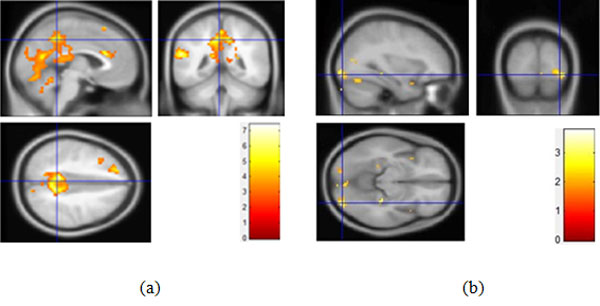
**Differences of activation caused by checkerboard stimulus between pre- and post- watching TV**. (a) watching 3DTV; (b) watching 2DTV.

The differences of activated regions obtained by checkerboard stimulus pre- and post-watching 3D/2DTV are mapped using two sample t-test. Compared to 2DTV group, the 3DTV group's result reveals statistically significant differences in frontal and subcutaneous besides temporal, parietal, occipital, limbic lobe. The 3D group results are shown in Figure 4a and Table 3 where R and L represent right hemisphere and left hemisphere individually. BA is a region in the brain cortex defined in many species based on its cytoarchitecture. K is the number of activated voxels. 2D group results are shown in Figure 4b and Table 4. From Table 3 and Table 4 the obvious differences exist in BA8, BA17, BA18, BA 19, BA32 and BA40. The phenomena imply that more functional areas of brain are involved to view 3D scenes than that of 2D scenes. As expected, checkerboard inductive stimulus have caused significant differences in visual cortex (BA17 and BA18) both in 2D group and 3D group, but the change of activated area in 3D group is greater than that of 2D group.

**Table 3:** Location of peak activation analysis of watching 3DTV.

Activation site(L/R)		MNI coordinate (mm)			BA	K	t value
					
		x	y	z			
**temporal lobe**	gyrus temporalis medius (R)	42	6	-27	38	21	7.4
	gyrus temporalis superior (R)	54	9	-18	38	105	4.33
	gyrus temporalis superior (L)	-36	9	-21	38	72	4.91
**parietal lobule**	precuneus (L)	-6	-42	45	18/19	331	6.78
	inferior parietal lobule (L)	-57	-45	21	40	39	6.65
	supramarginal gyrus (L)	-63	-51	27	40	42	4.29
**frontal lobe**	superior frontal gyrus (L)	-15	42	33	8/9	77	4.19
	superior frontal gyrus (R)	3	24	57		17	4.17
	middle frontal gyrus (L)	-30	33	30	8/9	109	5.04
	middle frontal gyrus (R)	42	45	15		32	5.24
	gyrus frontalis inferior (R)	38	11	-18	47	33	4.33
	cuneus (L)	-12	-75	9	17/18/23	48	3.36
	cuneus (R)	12	-87	21	18/19	22	3.82
**Subcutaneous**	caudate nucleus (R)	6	6	3		31	3.79
	lobus insularis (R)	45	-18	12	13	27	5.15
**temporal lobe**	anterior cingutate (L)	-6	21	21	24/32	396	5.54
	gyrus cinguli (R)	9	-45	42	23/29/30/31	147	6.43
	parahippocampal gyrus (L)	-24	-33	-15		15	3.37

Note: cluster size = 10, *p *< 0.01, uncorrected.

**Table 4:** Location of peak activation analysis of watching 2DTV.

Activation site(L/R)		MNI coordinate (mm)			BA	*K*	t value
					
		x	y	z			
**occipital lobe**	Inferior occipital gyrus(R)	33	-90	-9	18	32	3.17
	lingual gyrus(L)	-15	-90	-9	17/18	12	2.18
	cuneus(L)	-15	-99	-3	17	5	1.95
**parietal lobe**	inferior parietal lobule(L)	-54	-48	48	40	25	2.78
**limbic lobe**	anterior cingulate cortex(L)	0	21	21	32	13	2.22
**temporal lobe**	superior temporal gyrus(L)	-66	-36	18	40/42	13	2.27

Note: cluster size = 10, *p *< 0.01, uncorrected.

#### Differences of activation between watching 3D and 2D images

**Figure 5:**
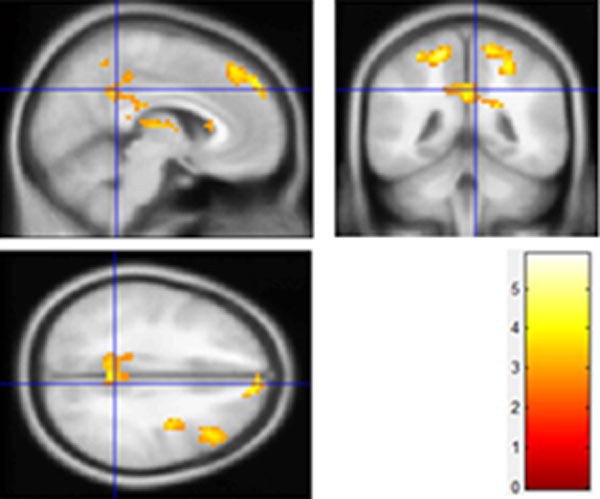
**Differences of activation between 3D and 2D images**.

Brain activation indicated the differences between watching 3D and 2D images in Figure 5 and Table 5. Compared to the stimuli task of 2D images, 3D images task have presented more significant relative activation in frontal lobe, limbic lobe as well as parietal lobe (p < 0.01). Specifically, there are stronger activations in superior frontal gyrus (R: 12, 48, 42, L: -21, 21, 57), middle frontal gyrus (L: -45, 18, 48, R: 24, 12, 48), cingulate gyrus (R:15, -39, 21), precentral gyrus (L: 0, -48, 33, R: 30, -15, 54), postcentral gyrus (L: -18, -42, 63, R: 21, -36, 60), superior parietal lobule (L: -18, -51, 60, R: 24, -51, 63), inferior parietal lobule (L: -33, -45, 60) thalamus(12, -15, 9) and dorsomedial nucleus (-6, -15, 9). Moreover, in terms of brain function, these regions mainly include general sensory area (BA3), somatosensory cortex (BA5, BA7), premotor cortex (BA6, BA8), frontal association cortex (BA19) and posterior cingulate cortex (BA31). This experiment demonstrates the relationship between the human brain activity and the stereoscopic stimulus.

**Table 5:** The more strongly activated sites produced by watching 3D images compared to 2D images.

Activation site(L/R)		MNI coordinate (mm)			BA	K	t value
					
		x	y	z			
**frontal lobe**	superior frontal gyrus (R)	12	48	42	8/9	161	5.78
	middle frontal gyrus (R)	24	18	48	8/9	292	5.34
	middle frontal gyrus (L)	-45	12	48	6/8	102	4.33
	superior frontal gyrus (L)	-21	21	57	8	30	3.86
**limbic lobe**	cingulate gyrus (R)	15	-39	21	31	136	5.45
**central gyrus**	precentral gyrus (L)	0	-48	33	31	23	3.99
	precentral gyrus (R)	30	-15	54	6/7	72	4.08
	postcentral gyrus (L)	-18	-42	63	3/5	68	4.93
	postcentral gyrus (R)	21	-36	60	3/4	70	3.81
**parietal lobule**	superior parietal lobule (L)	-18	-51	60	5/7	12	4.82
	inferior parietal lobule (L)	-33	-45	60	40	21	3.99
	superior parietal lobule (R)	24	-51	63	5/7	15	4.58
**Subcutaneous**	thalamus (L/R)	12	-15	9		94	4.28
	dorsomedial nucleus (L/R)	-6	-15	9		36	4.13

Note: cluster size = 10, *p *< 0.01, uncorrected.

#### Activated differences on the depth of field in front of and behind the focus point

**Figure 6:**
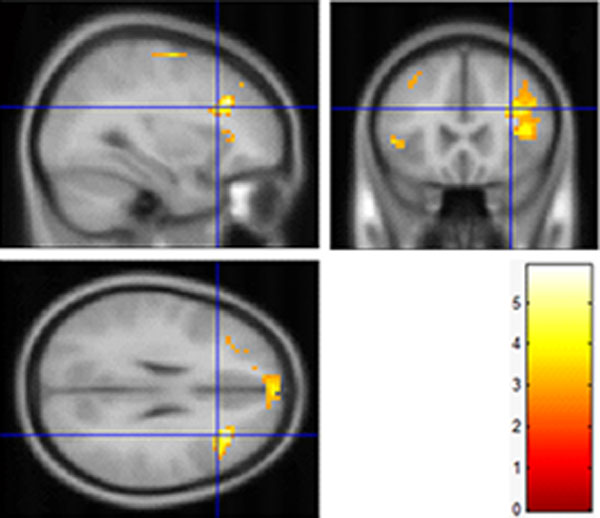
**Differences of activation as watching front and back depth of field images**.

In order to further investigate different effects of the depth of field front and behind the focus point on visual fatigue, the task of different image pattern were analyzed. And the results are revealed in Figure 6 and Table 6. Compared to the back depth of field task, the front depth of field task has shown greater activation in superior frontal gyrus (L: -30, 9, 60, BA8/9), middle frontal gyrus (L: -42, 9, 54, BA6/8/10, R: 39, 30, 33, BA6/8/9), inferior frontal gyrus (R: 45, 6, 33, L: -45, 21, 3), cingulate sulcus (R: 9, 9, 39, BA24/32, L: -3, 9, 39, BA24) as well as precuneus (R: 6, -54, 54, BA7). Thus it can add weight to the ideas that 3D display makes contributions to visual fatigue, and the front depth of field has more profound influences on visual fatigue.

**Table 6:** The more strongly activated sites produced by watching front depth of field images compared to that of the back one.

Activation site(L/R)		MNI coordinate (mm)			BA	K	t value
					
		x	y	z			
**frontal lobe**	middle frontal gyrus (L)	-42	9	54	6/8/10	138	5.87
	superior frontal gyrus (L)	-30	9	60	8/9	80	5.74
	middle frontal gyrus (R)	39	30	33	6/8/9	111	4.84
	gyrus frontalis inferior (R)	45	6	33		6	3.12
	gyrus frontalis inferior (L)	-45	21	33		34	3.65
	anterior central gyrus (R)	36	-18	58		20	3.28
**limbic lobe**	gyrus cinguli (R)	9	9	39	24/32	17	3.87
	gyrus cinguli (L)	-3	9	39	24	10	2.78
**parietal lobe**	precuneus (R)	6	-54	54	7	12	3.52

Note: cluster size = 10, *p *< 0.01, uncorrected.

## Discussion

As we know, there are significant increases in oxyhemoglobin and deoxyhemoglobin while watching TV. This should be attributed to the increase of cerebral blood flow (CBF) in response to the brain's energy demands. When the brain is activated in response to a visual task, the global CBF increases [17]. Conversely, when the metabolic rate became lower, such as under the mental fatigue, CBF decreases [18]. This is based on the fact that tissue ischemia increases the oxygen consumption, and thereby decreasing the venous oxyhemoglobin. Meanwhile, the decrease of venous oxyhemoglobin will lead to the reduction of BOLD signal.

Visual fatigue induced by the conflict between accommodation and convergence has already been reported in previous studies [19,20]. Based on the results of checkerboard stimulus performance before and after watching 3DTV or 2DTV, it has been testified that long time 3DTV watching will produce significant effects on brain function when subjects are required to process visual information in different depth. Compared directly to 2D group, the 3D group has shown more significant differences of brain activation, especially in BA8, BA17, BA18, BA19, BA32 and BA40. In these regions, activations of the visual cortices (BA17, BA18 and BA19) are associated with visual search [21]. Benjamin Backus et al. [22] have testified that occipital lobe (V3A, BA19) and parietal lobe (MT+, BA19) are sensitive to binocular disparity by fMRI experiments. There are obvious differences in occipital lobe (V3A, BA19) for stereoscopic task between macaque monkeys and human [23]. Beackus [22] has reported that BA19 (V3) is very sensitive to binocular vision disparity, and intimated with visual fatigue. Our research also demonstrates that there are huge differences in BA8, BA 17, BA18 and BA19. Other regions such as BA32 and BA40 indirectly associated with visual sense will not be discussed here. The changes of activated areas induced by checkerboard stimulus between pre- and post- 3DTV watching can be attributed to the fact that neuron activities are inhibited when the fatigue is increasing. After 1-hour watching, the level of excitement of checkerboard stimulus in 3D group is weaker than in 2D group. In consequence, the prolonged watching of 3DTV leads to more changes of activated areas than 2D group, which can be used to reflect the level of visual fatigue. In short, the response signals in BA8, BA17, BA18 and BA19 can be considered as main indicators to visual fatigue caused by watching 3DTV.

The frontal eye field (FEF, BA8) was confirmed to not only participate in eye movement, but also relate with stereoscopic depth perception [23]. From the result of activated differences between viewing 3D and 2D images, it has been testified that the premotor cortex (BA6 and BA8) has disparities because 3DTV displays a frame of video for the left eye and a frame of video for the right eye alternatively to form the stereoscopic impression. This lasting activation of BA8 is one of the reasons that watching 3DTV produces visual fatigue. Besides, the somatosensory cortex (BA5 and BA7) combining visual and movement information is responsible for the visual-movement coordination function in the brain. The results of this task performance imply that BA5, BA6, BA7 and BA8 are directly related to stereoscopic perception. Additionally, significant differences of activation have also been observed in general sensory area (BA3), frontal association cortex (BA9), and posterior cingulate cortex (BA31). These areas are in charge of sensory stimulation, memory and recognition capability respectively. Therefore, the BOLD signals in these areas can be used to report the level of mental fatigue occurred by prolonged watching of 3DTV, in which BA8 is directly related with vision.

While performing different depth of field stereoscopic image task, the result has shown that there are significant differences of brain activations in BA6, BA7 and BA8 between watching front depth field images and back depth of field images, especially in BA8. So, the front depth of field image could impose a more serious impact on visual fatigue than the back one.

## Conclusion

This study has investigated the relationship between human brain activity and visual fatigue induced by long time watching of 3DTV. The results are useful for healthy and reasonable 3DTV watching as well as properly designing of 3D scenes.

Although some valuable findings have been obtained, there are still several limitations in this study. After assuring that the experiment will not cause irreversible damage, especially for children whose visual system have not fully developed, more kinds of subjects should be selected to evaluate the visual fatigue brought by 3DTV watching. Additionally, some other physiological signals, such as EEG and EOG are needed to provide references along with fMRI acquisitions in the future.

## Competing interests

Other than the grant listed in the acknowledgement section, the authors declare that they have no other competing interest.

## Authors' contributions

C Chen is responsible for the experimental design and overall investigation, K Li, Y Liu and X Chen are responsible for doing experiments, including subjects recruitment and data collection. J Wang and K Li are responsible for statistical data analysis. All authors 1) have made substantial contributions to conception and design, or acquisition of data, or analysis and interpretation of data; 2) have been involved in drafting the manuscript or revising it critically for important intellectual content; and 3) have given final approval of the version to be published. Each author has participated sufficiently in the work to take public responsibility for appropriate portions of the content.

## Authors' information

C Chen' group has been doing research in the field of biomedical signal processing, and has published more than 40 papers in recent years.

Y Liu and X Chen are clinicians and have been working in the department of radiology for many years. They have rich experiences in terms of medical imaging analysis.

## Declarations

Publication of this article was paid with funding from National Natural Science Foundation of China (Grant No. 61171059).

This article has been published as part of *BioMedical Engineering OnLine *Volume 14 Supplement 1, 2015: Cardiovascular Disease and Vulnerable Plaque Biomechanics. The full contents of the supplement are available online at http://www.biomedical-engineering-online.com/supplements/14/S1
